# The Norwegian Cognitive impairment after stroke study (Nor-COAST): study protocol of a multicentre, prospective cohort study

**DOI:** 10.1186/s12883-018-1198-x

**Published:** 2018-11-26

**Authors:** Pernille Thingstad, Torunn Askim, Mona K. Beyer, Geir Bråthen, Hanne Ellekjær, Hege Ihle-Hansen, Anne Brita Knapskog, Stian Lydersen, Ragnhild Munthe-Kaas, Halvor Næss, Sarah T. Pendlebury, Yngve Muller Seljeseth, Ingvild Saltvedt

**Affiliations:** 1 Department of Neuromedicine and Movement Science, Faculty of Medicine and Health Science, NTNU Norwegian University of Science and Technology, Trondheim, Norway; 2 Department of Radiology and Nuclear Medicine, Oslo University Hospital, Oslo, Norway; 3 Department of Neurology and Clin. Neurophysiology, St Olavs University hospital, Trondheim, Norway; 4 Department of Internal Medicine, Stroke Unit, St. Olavs University Hospital, Trondheim, Norway; 5 Department of Geriatrics, Oslo University Hospital, Oslo, Norway; 6 Department of Medicine, Vestre Viken Hospital Trust, Bærum Hospital, Drammen, Norway; 7 Department of Geriatric Medicine, Oslo University Hospital, Oslo, Norway; 8 Department of Mental Health, Faculty of Medicine, Regional Centre for Child and Youth Mental Health and Child Care, NTNU - Norwegian University of Science and Technology, Trondheim, Norway; 9 Department of neurology, Haukeland University Hospital, Bergen, Norway; 10 Stavanger University Hospital, Stavanger, Norway; 11 Institute of clinical medicine, University of Bergen, Bergen, Norway; 12 Centre for Prevention of Stroke and Dementia, Nuffield Department of Clinical Neurosciences, University of Oxford, Oxford, UK; 13 NIHR Oxford Biomedical Research Centre, John Radcliffe Hospital, Oxford, UK; 14 Medical Department, Ålesund Hospital, Møre and Romsdal Health Trust, Ålesund, Norway; 15 Department of Geriatrics, St. Olavs University Hospital, Trondheim, Norway

**Keywords:** Post-stroke cognitive impairment, Dementia, Mild cognitive impairment, Cerebrovascular disorders, Stroke, Prevalence

## Abstract

**Background:**

Early and late onset post-stroke cognitive impairment (PCI) contributes substantially to disability following stroke, and is a high priority within stroke research. The aetiology for PCI is complex and related to the stroke itself, brain resilience, comorbid brain diseases, prestroke vulnerability and complications during the hospital stay. The aim of the Norwegian Cognitive Impairment After Stroke study (Nor-COAST) is to quantify and measure levels of cognitive impairments in a general Norwegian stroke population and to identify biological and clinical markers associated with prognosis for cognitive disorders following incident stroke. The study will be organised within five work packages: 1) Incidence and trajectories 2) Pathological mechanisms 3) Development of a risk score 4) Impact of physical activity and 5) Adherence to secondary prevention.

**Methods:**

Nor-COAST is an ongoing multicentre (five participating hospitals), prospective, cohort study with consecutive inclusion during the acute phase and with follow-up at three and 18 months, and at three years. Inclusion criteria are stroke defined according to the WHO criteria. During the recruitment period from 18.05.2015 to 31.03.2017, 816 participants have been included. Cognitive impairment will be classified according to the DSM-5 criteria using a consensus group. Cognitive function is assessed by a standardised neuropsychological test battery, the Montreal Cognitive Assessment, Trail making A and B, ten-word immediate and delayed recall test, the Controlled Oral Word Association, Global Deterioration Scale and proxy based information by and the Ascertain Dementia 8 item informant questionnaire. Biomarkers include magnetic resonance imaging, routine blood samples and bio-banking. Clinical assessments include characteristics of the stroke, comorbidity, delirium, frailty and tests for cognitive and physical function, sensor based activity monitoring and adherence to secondary prophylaxis.

**Discussion:**

Nor-COAST is the first Norwegian multicentre study to quantify burden of PCI that will provide reliable estimates in a general stroke population. A multidisciplinary approach aiming to identify biomarkers and clinical markers of overall prognosis will add new knowledge about risk profiles, including pre-stroke vulnerability and modifiable factors such as physical activity and secondary prophylaxis of relevance for clinical practice and later intervention studies.

**Trial registration:**

ClinicalTrials.gov: NCT02650531. Retrospectively registered January 8, 2016. First participant included May 18, 2015.

## Background

Stroke and cognitive impairment are common in old age. One in three persons will develop stroke, dementia or both [1], and the two conditions commonly coexist [2]. Impaired cognitive function contributes to the disability following stroke [3] and affects both the individual and the family and caregivers. With increased longevity and ageing population, stroke and dementia will constitute a substantial part of the disease burden in the years to come [4] with a potentially large socioeconomic gain when improving treatment and preventive strategies.

Cognitive function is therefore defined as a top priority for stroke research, especially studies of the links between cerebrovascular and degenerative disease are requested [5–7]. The term post-stroke cognitive impairment (PCI) is used to describe both mild cognitive impairment (MCI) and dementia that either become manifest three to six months after incident stroke (early-onset PCI) or develops over months and years (late-onset PCI). The observed delayed and accelerated decline in cognitive function after incident stroke suggest there is a therapeutic window for intervention and prevention of PCI [7]. Identification of biomarkers and clinical markers for risk of progression of PCI, could improve our understanding of the underlying pathological processes and help develop new strategies to reduce the burden of PCI.

### Incidence and trajectories of PCI

Reported rates for PCI varies considerably between studies. Many studies reports dementia but not MCI, and in studies that include MCI the operational definition has a large impact on the reported rate [8]. Pooled dementia rates suggest that 10% of first ever stroke survivors develop incident dementia within the first year, and 30% after recurrent stroke [9]. A Norwegian study reported an incidence of MCI and dementia one year after first ever stroke of 37.5 and 19.6%, respectively [10]. Heterogenity in reported rates of cognitive impairment following stroke can be explained by casemix differences resulting from population selection and attrition bias [6] and by varying assessment time and tools, operational definitions and diagnostic criteria [8]. Further there are few studies on the course of PCI, especially of late-onset PCI, and it is hypothesised that the course of PCI strongly depends on the underlying mechanisms [11–14]. To quantify the overall burden of PCI and explore distinct trajectories and their determinants, larger inclusive studies with longer follow-up are warranted.

### Biomarkers and predictors of PCI

A complex interplay of vascular pathology, neurodegenerative and inflammatory processes contributes to the progression of PCI [11, 14–16]. Cognitive impairment predisposes to stroke [15, 16] and patients with dementia get more severe strokes [17], supporting that the two conditions are related. Several imaging characteristics associated with PCI have been identified. Some imaging findings are more extensively studied, especially the relationship between white matter hyperintensities and cognitive impairment [18]. However, recently there has been more attention on the association between degenerative and vascular changes in the brain [2]. Chronic inflammation seems to be involved in the pathogenesis of both cerebrovascular disorders and Alzheimer’s disease. Immune activation and immune depression are present in the acute, sub-acute and chronic stage after a stroke and diminished or impaired inflammatory mechanisms are likely to be important factors in the pathways leading to dementia [19]. Cytokines are potential drivers in addition to neurovascular dysfunction, endothelial activation [20, 21] and genetic risk including Apolipoprotein Eε4 (APOE ε4) carrier status [22]. Several clinical markers are predictors of PCI, and the risk appears to increase with increasing number of predictors. Predictors include age, pre-stroke cognitive impairment, pre-stroke functioning and frailty as well as characteristics of the stroke lesion. In addition, complications in the acute and sub-acute phase as delirium, infections, falls and seizures seem to increase the risk [6].

Early and late-onset PCI is probably driven by somewhat different mechanisms, with brain resilience and characteristics of the stroke lesion being the dominant driver for early-onset PCI, while small vessel disease, Alzheimer pathology and recurrent strokes are more pronounced in late-onset PCI [11, 14, 23, 24].

### Secondary prevention, adherence and physical activity

Prevention of recurrent strokes is important in preventing PCI [6] and can probably be achieved in up to 80% by optimal secondary prevention [25]. A multi domain intervention including vascular risk monitoring and physical activity has shown to prevent cognitive decline in at-risk stroke free elderly people suggesting that secondary prevention could have an additional and independent effect on cognition and overall brain health [26]. However, guidelines for secondary prevention are rather complex, involving secondary preventive medications and healthy lifestyle including smoking cessation, moderate alcohol consumption, healthy diet and regular physical activity [27]. Adherence to both medication and lifestyle interventions have been reported to be suboptimal in many patients [28], and adherence to secondary prevention may be problematic in patients with cognitive impairment after stroke.

There is evidence that physical activity enhances cognitive function after stroke [29], and especially executive function in individuals with MCI [30]. A recent meta-analysis, representing data from 736 participants included in 14 randomized controlled trials, demonstrated a significant positive effect of physical activity on cognition post-stroke [31]. Possible mechanisms involved are reduced levels of inflammation [32], improved brain circulation, neurogenesis and increased size of hippocampus [33, 34]. On the other hand, sedentary behaviour has been identified as a risk factor for vascular disease, and hypothesized to increase the risk of cognitive impairment after stroke [35].

### Study aims

The aim of the Norwegian Cognitive Impairment After Stroke study (Nor-COAST), is to quantify and measure levels of cognitive impairments in a Norwegian general stroke cohort and to identify biomarkers and clinical markers associated with overall prognosis for early and late onset PCI.

**Fig. 1:**
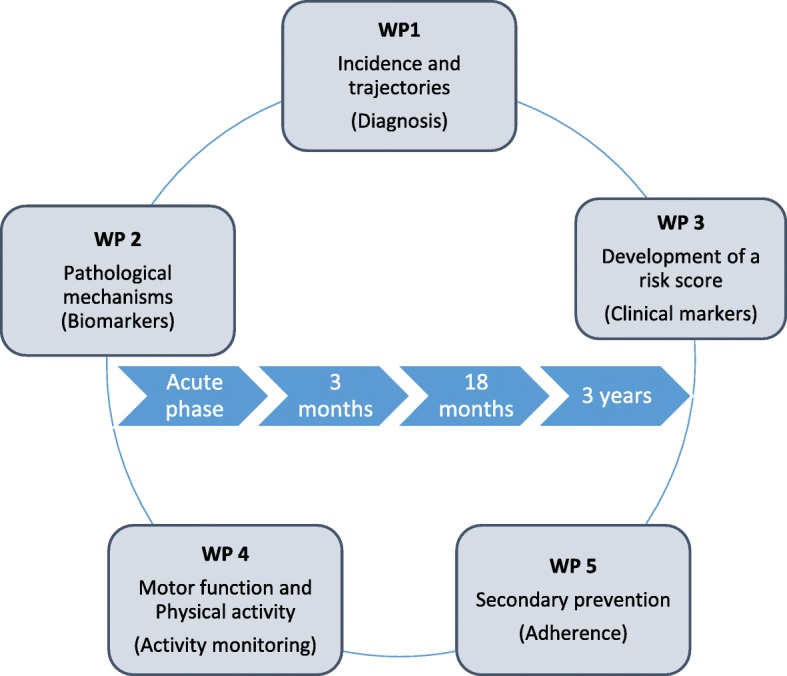
Nor-COAST is organised within five work packages (WP)

The Nor-COAST study is organised within five work packages (WP). The aim of WP 1 is to describe incidence and distinct trajectories of PCI. In WP 2, the aim is to increase knowledge concerning the pathogenesis of early and late-onset PCI by analysing brain imaging, APOE ε4 typing and blood markers, and to explore associations between neurodegenerative disease, underlying vascular disease, inflammation and stroke characteristics. In WP 3, we aim to identify predictors for PCI in order to develop a novel risk score consisting of a few clinical markers that will enable planning of individualized prevention and treatment. The aim of WP 4 is to study the impact of physical activity and sedentary behaviour on PCI in the short and long term, and to explore associations between motor function measured by performance based tests and progression of PCI. WP 5 aims to explore the impact of cognitive function on adherence to secondary prevention and evaluate whether optimal secondary prophylaxis is protective against decline in cognitive function over time Fig. 1.

## Methods/design

The Nor-COAST study is a multicentre, prospective, cohort study following participants for three years from the stroke incident. Recruitment started successively at the different sites between 18.05.2015 and 01.09.2016 and was ended as preplanned 31.03.2017, with 816 participants included. Participants were recruited from five Norwegian stroke units at the following hospitals; St. Olavs University Hospital, Trondheim (*n* = 400); Ålesund Hospital, Møre and Romsdal Health Trust, Ålesund (*n* = 33), Haukeland University Hospital, Bergen (*n* = 142); Bærum Hospital, Vestre Viken Hospital Trust, Drammen (n = 142) and Oslo University Hospital, Ullevål, Oslo (*n* = 99). All patients admitted to the participating stroke-units were consecutively screened for eligibility and approached as soon as the diagnosis was confirmed. Informed consent was given prior to data collection by the participant or by proxy if the participant lacked capacity to give informed consent. All participants received treatment and routine follow-up as usual in accordance with national and international guidelines [36, 37].

### Sample size

Based on the Norwegian stroke registry we estimated that about 1000 stroke patients in total would be available for inclusion per year in the participating hospitals. Experience from earlier studies at the same hospitals involved [10, 38] indicated that about half of eligible participants would be included and that 20–25% would be lost to follow-up during the data collection period of 18 months. With 750 patients available for follow-up, we will be able to detect an association corresponding to a correlation of 0.11 or higher (power 86% at significance level 5%). Inclusion of approximately 1000 participants would therefore be sufficient to answer the main research questions within each work package. Based on these assumptions we decided on a preplanned inclusion stop at 1000 participants or a maximum of two years inclusion period.

### Participants

Eligibility criteria are: 1) Admittance to one of the five centres within seven days after symptom debut. 2) Acute stroke diagnosed according to the World Health Organisation (WHO) criteria or with findings of acute infarction or intra-cerebral haemorrhage on MRI. 3) Scandinavian speaking 4) over > 18 years and 5) living in the catchment area of the recruiting hospitals. Exclusion criteria were expected survival less than three months.

### Data collection and assessments

Assessments during the hospital stay are performed at discharge or the seventh day of the stay for participants with longer hospital stay. Study related follow-ups are performed at the outpatient clinic three and 18 months, and three years’ post-stroke. Participants unable to attend the outpatient clinic are assessed by telephone interview and/or proxy information is collected. Assessments and interviews are performed by trained research assistants, using a standardised paper Case Report Form (CRF), and then plotted in web-based CRF developed by the Unit for Applied Clinical Research, NTNU. Table 1 provides an overview of data collected by time and data source.

**Table 1:** Overview of assessments, source of data and time of assessment

T0: Baseline, T1: Three months follow-up, T2: 18 months follow-up, T3: Three years follow-up	Medical records	Examination	Questionnaire	Proxy	T0	T1	T2	T3
Demographics	x		x		✓	✓	✓	✓
Health history			x	x	✓	✓	✓	✓
Medication	x		x	x	✓	✓	✓	✓
Magnetic Resonance Imaging		x			✓		✓	✓
Clinical blood samples		x			✓	✓	✓	✓
Biobanking		x			✓	✓	✓	✓
Stroke characteristics								
National Institute of Health Stroke Scale – NIHSS		x			✓	✓	✓	✓
Oxfordshire Stroke /TOAST classification	x				✓			
Activities of Daily Living (ADL)								
Modified Rankin Scale (mRS)		x	x	x	✓	✓	✓	✓
Barthel Index		x	x	x	✓	✓	✓	✓
Nottingham E-ADL			x	x	✓	✓	✓	✓
Cognitive function								
Montreal Cognitive Assessment (MoCA)		x			✓	✓	✓	✓
Trail making Test A + B					✓	✓	✓	✓
Ten-word immediate and delayed recall test (CERAD)		x				✓	✓	✓
The Controlled Oral Word Association Test (COWAT)		x				✓	✓	✓
Global Deterioration Scale (GDS)		x	x	x	✓	✓	✓	✓
The Ascertain Dementia 8-item Informant Questionnaire (AD8)			x	x	✓	✓	✓	✓
The Confusion Assessment Method (4-item CAM)	x				✓			
Mental function								
Hospital Anxiety and Depression Scale (HADS)			x			✓	✓	✓
Cornell Scale for depression in Dementia			x	x		✓	✓	✓
Neuropsychiatric Inventory Questionnaire (NPI-Q)			x	x		✓	✓	✓
Line bisection neglect test		x			✓	✓	✓	✓
Health Related Quality of Life								
EQ-5d-5 L			x			✓	✓	✓
Physical function								
Short Physical performance Battery (SPPB)		x			✓	✓	✓	✓
10 m gait speed		x			✓	✓	✓	✓
Dual task gait (counting backwards)		x			✓	✓	✓	✓
360 degrees		x			✓	✓	✓	✓
One-leg stand		x			✓	✓	✓	✓
Nine-hole peg		x			✓	✓	✓	✓
Grip strength (Jamar)		x			✓	✓	✓	✓
Physical Activity								
ActivPal (accelerometer)		x			✓	✓	✓	✓
Frailty								
FRAIL Scale			x		✓	✓	✓	✓
Fatigue Severity Scale (FFS)			x			✓	✓	✓

### Outcomes and measurements

#### Demographics, health history and disability

Sociodemographic characteristics are registered based on medical records and interviews with patients and/or caregivers. Information of health history, premorbid function and life style habits are recorded using standardised questionnaires from the Norwegian stroke registry and the North-Trøndelag Health (HUNT) Study. Charlson comorbidity index identifies comorbidity, [39] and a measure of frailty is achieved by the FRAIL scale [40]. The Modified Rankin Scale (mRS) evaluates the global function, and instrumental activities of daily living (I-ADL) is assessed by the Nottingham Extended ADL scale [41] with the 4 subdomains of mobility, leisure time activity, kitchen and domestic work. For basic ADL, the Barthel Index [42] is used assessing independence in 10 basic ADL functions. Health related quality of life is assessed using EQ-5D-5 L, [43] and fatigue by the seven-item version of the Fatigue severity scale (FSS-7) [44].

#### Stroke characteristics, complications and medical treatment

Stroke symptoms and severity is assessed by National Institutes of Health Stroke Scale (NIHSS). Strokes are classified according to the Oxfordshire Stroke Classification Project (OSCP) [45] and the TOAST classification [46]. The confusion assessment method (CAM four item version) for delirium evaluation is performed by the nurses the first two days of hospital stay [47]. Acute treatment, including thrombolysis and thrombectomies is obtained from medical records. Use of medication at the time of admission and at discharge is recorded based on information from participants, proxies and medical records. All drugs are classified by using the ATC-classification system. Evaluation of the adherence to medication at three and 18 months are based on self-reports according to the 4-item Morisky Medication Adherence Scale (MMAS-4) [48]. Follow-up routines in primary health care will be investigated in a substudy.

#### Neuropsychological assessment

MCI and dementia are diagnosed according to the DSM-5 criteria [49], an expert panel will make consensus on how to classify the cases. The neuropsychological test battery in Nor-COAST is based on recommendations from the National Institute of Neurological Disorders and Stroke-Canadian Stroke Network (NINDS-CSN) using the medium, 30 min version [50], including Trail making A and B (TMT A and B) [51], ten word memory and recall test (CERAD) [52] and the controlled oral word association test (COWAT) [53].

Screening of global cognitive function is performed using the Montreal Cognitive Assessment (MoCA) [54]. MoCA evaluates several cognitive domains like memory, visuospatial abilities, executive function, language, abstraction, attention, subtraction, digits forward and backward and orientation and is also validated for telephone interview [55]. Proxy based information on cognition is collected using the Ascertain Dementia 8-item informant questionnaire (AD-8) [56]. The Global Deterioration Scale (GDS) [57] measures global cognition and is scored by the assessor, based on the combination of available information from tests and interviews.

Neuropsyciatric Inventory (NPI-Q) [29] is a 12-item questionnaire assessing occurrence and severity of observed neuropsychiatric symptoms based on self-report or proxy information. Symptoms of anxiety and depression are screened for using Hospital Anxiety and Depression Scale (HADS) [58] for self-reported symptoms. If the participants are not able to respond, proxy information on depression is collected by the Cornell scale [59].

#### Physical function and activity monitoring

Lower extremity function is measured by Short Physical Performance Battery (SPPB) consisting of three subtasks: 4-m gait speed, balance and chair rise [60]. Additional balance tests include one-leg-stance and 360* turning [61]. Grip strength is measured by using a Jamar handhold dynamometer. The nine-hole peg test is used to measure dexterity [62]. Gait speed at preferred and fast speed, and during dual task-performance (counting backwards) are measured across 10 m with flying start.

Physical activity is measured using a three-axial accelerometer, ActivePAL monitors (PAL Technologies Ltd., Glasgow, United Kingdom), attached to the front of the unaffected thigh during the hospital stay and for a minimum of four days at three and 18 months’ and three years’ follow-up. The inertial sensor produces a signal related to thigh inclination and can thus identify posture (sitting/lying from upright activity) [63]. This method is shown to be valid in the stroke population [64].

#### Blood samples

Blood samples are analysed during the index stay and at three and 18 months and three years for haemoglobin, low density lipoprotein (LDL), high density lipoprotein (HDL) and total cholesterol, glucose, glycosylated haemoglobin (HbA1c), creatinine, high sensitive CRP and troponin T. In addition, blood has been sampled in BioBank 1, Central-Norway Regional Health Authority, at baseline, three and 18 months and three years for analyses regarding genetics and inflammation. If full blood can not be sampled, saliva is collected in order to make genetic analyses (APOE ε4 carrier status).

#### Neuroimaging

All patients undergo an acute CT scan to establish the stroke diagnosis and the majority have a brain MRI scan as part of routine diagnostic work-up. In addition, participants are recruited for a study specific MRI brain scan within the first 14 days after stroke. The MRI study protocol consists of 3D-T1, axial T2, 3D-FLAIR, DWI and SWI sequences. A follow-up scan with identical MRI protocol is performed after 18 months and at three years. Protocols were harmonized between centres after scanning of the same healthy volunteer and stroke volunteer in all scanners.

A standardized assessment of the MR findings includes systematically scoring for global atrophy, focal atrophy and vascular disease. For evaluation of white matter hypertensities (WMH) we will use the Fazekas scale [65]. For parietal atrophy we will use the posterior atrophy scale [66]. Lacunes and cortical infarcts will be registered and microbleeds will be assessed using the Bombs scale [67]. Assessment of medial temporal lobe atrophy (MTA) will be done using the Scheltens scale [68]. We will follow the suggested STandards for ReportIng Vascular changes on nEuroimaging (STRIVE) [69]. Quantitative group analysis of the study specific MRI with automated and semi-automated techniques will also be performed.

#### National registries

Additional information on diagnosis, treatment, and cause of death will be obtained from the Norwegian Stroke Register, the Norwegian cardiovascular disease registry, the Norwegian Prescription Database, the Norwegian Patient Registry and the Norwegian cause of death registry. Administrative permission to access data from registries will be obtained within each workpackage according to the specific research questions.

### Statistical analysis

Demographics and participant characteristics will be presented using descriptive statistics. Prevalence and incidence of dementia will be presented by crosstabs. Statistical analyses for trajectories will be performed with group-based trajectory modelling, which uses maximum likelihood to identify groups of individuals with statistically similar trajectories Two primary outputs of the estimations in group-based trajectory modelling are 1) shapes of trajectories of each group as specified by group-specific polynomial functions of time and 2) estimated percentages of the population following each identified trajectory [70]. Both results from visual and volumetric brain analyses will be used to identify MRI predictors of early or delayed of cognitive impairment after stroke. Brain MRI analyses will be combined with machine learning techniques to explore eventual clusters in the study population and to build predictive models. Network analysis with the “NetworkX” software package [71]. is used to discover patient clusters. Predictive models are build with classification algorithms like decision trees with “scikit-learn” [72]. and gradient boosting with “XGBoost” [73]. Linear regression, logistic regression and other statistical methods will be used according to the specific research questions within each work package. A statistician is part of the project managing group and will be involved in developing analysis plans for the main publications and quality insurance of statistical approach in each work package.

## Discussion

The current paper describes the protocol of the Nor-COAST study, an ongoing prospective, cohort study following 816 participants for three years after incident stroke. This will be the first study to quantify and report levels of cognitive impairment in a large Norwegian stroke cohort. The study will focus on the diagnosis, incidence and clinical entity of PCI, and provide estimates for the burden of both early and late onset PCI in a general stroke population.

The neuropsychological test battery used in the study is sensitive to the mild cognitive impairments including non-amnestic deficits [74] that are common in the stroke population. This will be among the first studies to use the DSM-5 criteria to diagnose MCI and dementia. In contrast to the ICD-10 criteria, the DSM-5 criteria do not require memory deficit for PCI diagnosis and are consistent with the VASCOG criteria for diagnosing vascular cognitive disorders [75] . Three years’ follow-up will allow study of the mechanisms for late-onset PCI for which there are currently few data.

Using registry data from the Norwegian stroke registry allow us to account for some of the methodological challenges associated with bias, drop-outs and testability in a heterogenic population [76–78]. This along with collection of proxy based information and systematic registrations of reason for missing data should be an advantage compared to many earlier studies aiming to describe the overall burden of PCI.

By neuroimaging and identification of genetic, vascular and inflammatory response-factors, we hope to achieve insight of stroke as a trigger for degenerative brain processes and the relevance of pre-existing risk factors, including brain pathologies, for development of PCI. Both results from visual and volumetric brain analyses will be used to identify MRI predictors of early or late onset cognitive impairment. The longitudinal design of Nor-COAST enables us to look for the contribution of vascular and degenerative changes to delayed onset PCI, and to study the characteristics of patients with augmented and prolonged inflammatory response.

We aim to develop a reliable and accurate tool to identify patients at risk of developing PCI based on clinical markers in early phase after stroke. This will provide insight into the relative importance of patient vulnerability in terms of frailty, comorbidity, pre-stroke function and cognition, and the relation between cognitive outcome and stroke and non-stroke factors such as location and severity of stroke, specific treatment and complications including delirium. Further, we aim to explore whether inclusion of biomarkers will increase the predictive value of the score. Identifying stroke survivors at high risk at an early stage would enable early, personalized and appropriate intervention for prevention.

Physical activity will be measured by use of body worn sensors. This will make it possible to monitor physical activity continuously for up to one week at inclusion and at each follow-up, and thereby avoid some of the methodological challenges, such as recall bias and overestimation of activity, associated with the most commonly used self-reported measures [79–82]. These data will give the opportunity to investigate the underlying mechanisms of the possible preventive effect of physical activity or the negative effects of sedentary behaviour on early and late onset of PCI. Identifying characteristics associated with sedentary behaviour could further help targeting individuals who might benefit from closer follow-up. Gait speed, lower extremity function, balance and dexterity are assessed during the acute stay and at each follow-up and will allow us to explore how different aspects of motor function are associated with and predict PCI. There are studies indicating that screening of motor function early after stroke might help identify individuals at risk of PCI [83], however, larger studies are required.

The concept of adherence, will be assessed by combining information from subjective and objective sources. With supplementary information and evaluation of follow-up routines in primary health care, this sub-study constitutes a valuable approach to a challenging and sparsely studied research area where further data are warranted [84, 85]. Secondary prevention is important for the prognosis following a stroke, both because the risk of PCI is strongly increased by a subsequent stroke, but also because optimal treatment of vascular risk factors seem to impact neurodegeneration and small vessel disease. The Nor-COAST study, collecting crucial data for three years after a stroke, will give unique insight into how PCI influences adherence to preventive measures and achievement of treatment goals and secondly influences the risk of stroke and PCI.

The results of our comprehensive approach, combining assessment of biomarkers and clinical markers along with modifiable factors including physical activity and adherence, holds potential to reveal knowledge that could inform routine clinical practice and be easily applicable in clinical practice. We aim to develop a tool for the early identification of persons at risk and to improve knowledge about modifiable factors such as physical activity and secondary prophylaxis important for prevention, treatment, rehabilitation and overall prognosis. Increased knowledge of adherence to a healthy lifestyle and pharmacological treatments will enable us to give realistic and feasible individualised advice to prevent recurrent stroke and PCI.

Combination of biomarkers and clinical markers allow us to explore the relationship between inflammation and APOE status, chronic inflammation, sedentary behaviour, stroke recurrence and cognition. Brain MRI analyses will be combined with advanced statistical techniques to find combinations of MRI findings and clinical variables associated with the development of PCI. The multidisciplinary approaches applied in the present study unite dementia, geriatrics, stroke and physiotherapy research. This gives us an opportunity to extend existing perspectives for cooperation and to exploring the impact of for example frailty and neurodegeneration on long-term outcome after stroke.

There are some limitations of the Nor-COAST study. Although the neuropsychological test battery contains most of the cognitive domains in the DSM 5, symptoms related to behavior and social cognition are not extensively assessed. Further, some selection bias may have occurred despite the inclusive nature of the study: potential participants may not have been included because of worse health status. Also the recommended neuropsychological battery is cited as taking up to 30 min to perform, but many patients needed longer or testing had to be abandoned. In addition, some assessments, eg the MRI study and parts of the cognitive testing, were not feasible for all participants because of severe illness or specific impairments.

## Summary and conclusions

The Nor-COAST study will provide reliable estimates of the burden of PCI in a general Norwegian stroke population. Further, the study will add new and important knowledge on the early detection of individuals at risk for progression of PCI, extend understanding of the underlying pathophysiology and the impact of pre-stroke vulnerability, early physical activity and adherence to secondary prevention on prognosis. The results will contribute to the development of new guidelines for routine clinical practice in stroke and better understanding of mechanisms underlying PCI allowing the development of more specific hypotheses and the design of future intervention studies.

## Abbreviations

3D-FLAIR: Three-dimensional Fluid-attenuated inversion recovery (FLAIR)
AD8: Ascertain Dementia 8-item informant questionnaire
APOE: ε4 Apolipoprotein Eε4
ATC: Anatomical Therapeutic Chemical (ATC) Classification System
CAM: The Confusion Assessment Method
CERAD: Consortium to Establish a Registry for Alzheimer’s Disease
COWAT: Controlled Oral Word Association Test
CRF: Standardised case report form
CRP: C-reactive protein
CT: Computer Tomography
DSM-5: The Diagnostic and Statistical Manual of Mental Disorders, Fifth Edition
DWI: Diffusion-weighted magnetic resonance imaging
EQ5D-5 L: The five level EuroQol five dimension
FSS-7: Seven Item Fatigue Severity Scale
GDS: The Global Deterioration Scale
HADS: Hospital Anxiety and Depression Scale
HbA1c: Glycosylated haemoglobin
HDL: High density lipoprotein
HUNT: Population based health survey in North-Trøndelag
I-ADL: Instrumental Activities of Daily living
LDL: Low Density Lipoprotein
MCI: Mild Cognitive Impairment
MMAS-4: 4-item Morisky Medication Adherence Scale
MoCA: Montreal Cognitive Assessment
MRI: Magnetic Resonance Imaging
mRS: Modified Rankin Scale
MTA: Medial temporal lobe atrophy
NIHSS: National Institutes of Health Stroke Scale
NINDS-CSN: The National Institute for Neurological Disorders and Stroke and Canadian Stroke Network
Nor-COAST: Norwegian Cognitive impairment After STroke study
NPI-Q: Neuropsyciatric Inventory
OSCP: Oxfordshire Stroke Classification Project
PCI: Poststroke cognitive impairment
SPPB: Short Physical Performance Battery
STRIVE: STandards for ReportIng Vascular changes on Euroimaging
SWI: Susceptibility weighted imaging
TMT A and B: Trail making A and B
TOAST: The Trial of Org 10,172 in Acute Stroke Treatment
WMH: White Matter Hypertensities
WP: Work Package
